# Faecal bacterial composition in horses with and without free faecal liquid: a case control study

**DOI:** 10.1038/s41598-021-83897-4

**Published:** 2021-02-26

**Authors:** Katrin M. Lindroth, Johan Dicksved, Erik Pelve, Viveca Båverud, Cecilia E. Müller

**Affiliations:** 1 Department of Animal Nutrition and Management, Swedish University of Agricultural Sciences, 750 07 Uppsala, Sweden; 2 Department of Anatomy, Physiology and Biochemistry, Swedish University of Agricultural Sciences, 750 07 Uppsala, Sweden; 3 National Veterinary Institute, 751 89 Uppsala, Sweden

**Keywords:** Animal physiology, Microbial communities, Dysbiosis

## Abstract

Free faecal liquid (FFL) is a condition in horses which manifests as differential defecation of solid and liquid phases of faeces. The etiology of FFL is currently unknown, but deviances in the hindgut microbiota has been suggested to be of importance. The present study aimed to compare the faecal bacterial composition of farm-matched horses with (case, n = 50) and without (control, n = 50) FFL. Samples were collected at three different occasions. The V3 and V4 regions of the 16S rRNA gene were amplified and sequenced using Illumina sequencing. Also, samples were cultivated for detection of *Clostridioides difficile* and *Clostridium perfringens*. Analysis revealed similar faecal bacterial composition between case and control horses, but an effect of sampling period (p = 0.0001). Within sampling periods, 14 genera were present in higher or lower proportions in case compared to control horses in at least one sampling period. Compared to controls, case horses had higher relative abundance of *Alloprevotella* (adjusted p < 0.04) and lower relative abundance of *Bacillus* spp*.* (adjusted p < 0.03) in at least two sampling periods. All horses tested negative for *C. difficile* and *C. perfringens* by culture of faeces. Further studies are required to establish the clinical relevance of specific bacterial taxa in FFL.

## Introduction

Free faecal liquid (FFL), also referred to as faecal water syndrome (FWS), in horses manifests as differential solid and liquid phases at defecation, where the liquid phase is voided before, during or after defecation of the solid phase or completely separate from the solid phase. The condition may last from a few days to months and sometimes years, and it may vary in severity over time^1–4^. Causes of FFL in horses are unknown^4^. Several factors have been suggested to be implicated in FFL, such as endoparasitic infection, poor dentition resulting in insufficient chewing of feed, the horse being over 20 years old, feeding high amounts of alfalfa (*Medicago sativa*) and feeding wrapped forages as silage or haylage instead of hay^3^, but no clear relations to these factors have been identified^1,3–5^. Results from a survey of FFL-horses showed that the studied horse population had a comparably high incidence of colic^4^ compared to other horse populations. It has been shown that horses with colic^6^ and diarrhoea^7^ have a different faecal microbiota composition compared to healthy controls, which may also be the case for horses with FFL. Diarrhoea in horses has previously been associated to intestinal overgrowth of *Clostridial* species^8^, but so far no enteric pathogen has been linked to FFL. Due to this, and to the nature of FFL resembling chronic diarrhoea, it is of interest to evaluate the role of hindgut microbiota composition as well as presence of *Clostridial* species associated with diarrhoea in horses with FFL. Minor differences in faecal bacterial composition in horses with and without FFL have been reported^9^ indicating that specific taxa may be of importance and interest for the presence of FFL in horses. The objective of this study was therefore to compare the faecal bacterial composition in horses with and without FFL, in order to gain further knowledge about this condition.

## Results

Basic demographics and clinical characteristics of case and control horses. There were no differences (p < 0.05) between case and control horses for the basic demographic variables reported in Table 1. Case horses were reported by their owners to have a faecal appearance score (FAS) of 4–6 and the majority (54%) were reported to have a FAS of 4. Control horses were reported by their owners to have FAS of 1–3 and the majority (67%) were reported to have a FAS of 2 (Table 2). No difference in FAS was found between horse owner assessments and assessment by the evaluator at the laboratory (p > 0.05). All horses tested negative for *Clostridioides difficile* and *Clostridium perfringens* by culture of faeces. Nine samples, from nine different horses (six case and three control horses, including two case–control pairs) tested positive for *C. perfringens* after enrichment of faecal samples.

**Table 1:** Basic demographics of case horses with (n = 50) and control horses without (n = 50) free faecal liquid included in the study.

Variable	Case, average (SD)	Control, average (SD)
Age (years)	13 (5.67)	10 (5.28)
Variable	Case, n (%)	Control, n (%)
**Gender**		
Gelding	27 (54)	22 (44)
Mare	23 (46)	28 (56)
**Breed type**		
Warmblood horses	21 (42)	19 (38)
Cold-blood horses	14 (28)	8 (16)
Thoroughbreds	4 (8)	3 (6)
Ponies	11 (22)	20 (40)
**Body Condition Score (BCS)**^a^		
BCS < 3	11 (22)	5 (10)
BCS = 3	26 (52)	33 (66)
BCS > 3	13 (26)	12 (24)

^a^Body condition score according to Carroll and Huntington^10^, assessed by the horse owner. Case and control groups had similar proportions of horses for all variables (P > 0.05).

**Table 2:** Faecal appearance score (FAS; 1–7) for faecal samples from case horses with free faecal liquid (case) and without free faecal liquid (control), for sampling periods (SP) 1–3 assessed by the horse owners.

Faecal Appearance Score (FAS)	SP1		SP2		SP3	
	Case, n (%)	Control, n (%)^a^	Case, n (%)	Control, n (%)	Case, n (%)	Control, n (%)
1	0 (0)	3 (6)	0 (0)	2 (5)	0 (0)	4 (7)
2	0 (0)	33 (67)	0 (0)	34 (68)	0 (0)	38 (76)
3	0 (0)	13 (27)	0 (0)	14 (27)	0 (0)	8 (17)
4	26 (54)	0 (0)	28 (56)	0 (0)	29 (58)	0 (0)
5	12 (23)	0 (0)	12 (23)	0 (0)	14 (27)	0 (0)
6	12 (23)	0 (0)	10 (21)	0 (0)	7 (14)	0 (0)
7	0 (0)	0 (0)	0 (0)	0 (0)	0 (0)	0 (0)

^a^n = 49. Case and control groups had similar proportions of horses for all FAS (P > 0.05).

The majority of both case and control horses were reported to have a parasitic burden below detection level (< 50 eggs per gram, EPG) according to national guidelines (www.sva.se) for tapeworm (*Anoplocephala perfoliata)* (64% of all case and control horses), small strongyles (*Cyathostomins)* (88% of all case and control horses), large strongyles (*Strongylus vulgaris)* (100%) and roundworm (*Parascaris* spp.) (100%). There was no difference in faecal egg count between case and control horses (p > 0.05). Detailed information on parasitic burden is presented in Supplementary Table S1. None of the horses was reported to have had pyrexia (≥ 38.3 °C) at the time of sampling and no difference in rectal temperature between case and control horses was observed (p > 0.05). Detailed information on rectal temperatures for all horses are presented in Supplementary Table S2.

**Figure 1:**
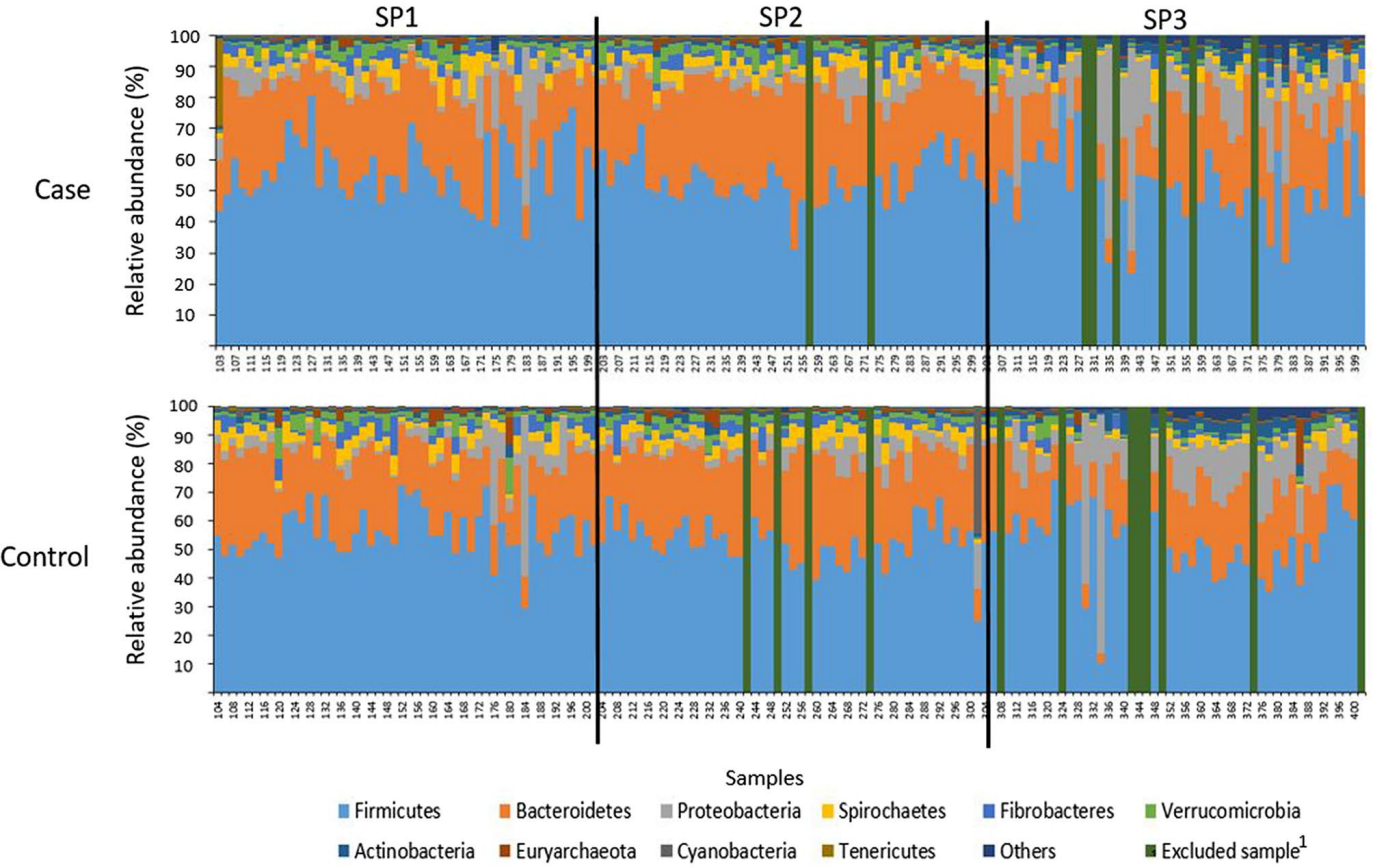
Bar chart illustrating variation in relative abundance of the 10 most abundant bacterial phyla in faecal samples from horses with free faecal liquid (case, n = 142) and horses without free faecal liquid (control, n = 138), for sampling periods (SP) 1–3. Sampling periods are separated by black vertical lines. Samples from case and control horses within each pair are located vertically in the bar chart for comparisons within each horse pair. ^1^Green bars represents samples that were excluded for not passing the quality control.

**Figure 2:**
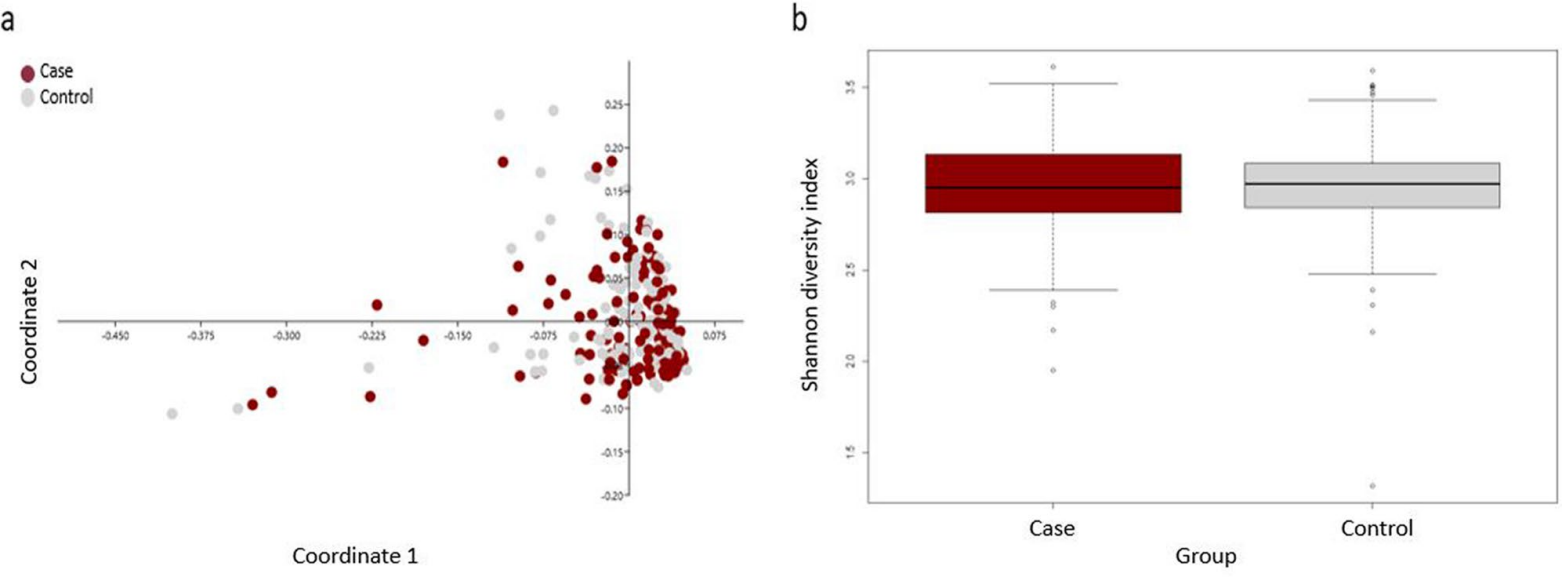
Principal coordinate analysis (PCoA) with Bray–Curtis similarity (**a**) and boxplot of Shannon diversity (**b**) for horses with free faecal liquid (case, n = 142) and without free faecal liquid (control, n = 138).

Faecal bacterial composition in case and control horses. In total, 280 samples (142 and 138 samples from case and control horses, respectively) were included in the final analysis. Twenty (7.0%) samples (case: n = 8 and control: n = 12) did not pass the quality control prior to sequencing and therefore no data was retrieved from these samples. Results from the 280 faecal samples sequenced gave a total count of 25,068,459 reads, with an average of 88,782 ± 8863 high quality sequences (mean ± s.d.) read per sample for case horses and 89,012 ± 8486 (mean ± s.d.) reads per sample for control horses. The relative abundance of the 10 most abundant phyla for case and control horses is presented in Fig. 1, illustrating individual difference in proportions of the top 10 phyla for all horses. For both case and control horses, *Firmicutes* was the most prevalent phylum (average 54.0% and 53.8%, respectively), followed by *Bacteroidetes* (average 26.2% and 25.3%, respectively) and *Proteobacteria* (average 7.8% for both case and control). On genus level, the most abundant for both case and control horses was *Rikenellaceae RC9 gut group* (average 4.9% and 5.1%, respectively), followed by *Solibacillus* (average 3.3% and 4.7% of reads, respectively) and *Treponema 2* (average 3.2% and 3.4%, respectively). Faecal microbial composition was compared between case and control horses using PCoA. This did not reveal any separate clustering between case and control horses (Fig. 2a), which was also confirmed in the ANOSIM analysis (*p* = 0.21; R = 0.003). Neither were there any differences in number of observed species (p = 0.23) or Shannon diversity (p = 0.79) between case and control horses (Fig. 2b).

**Figure 3:**
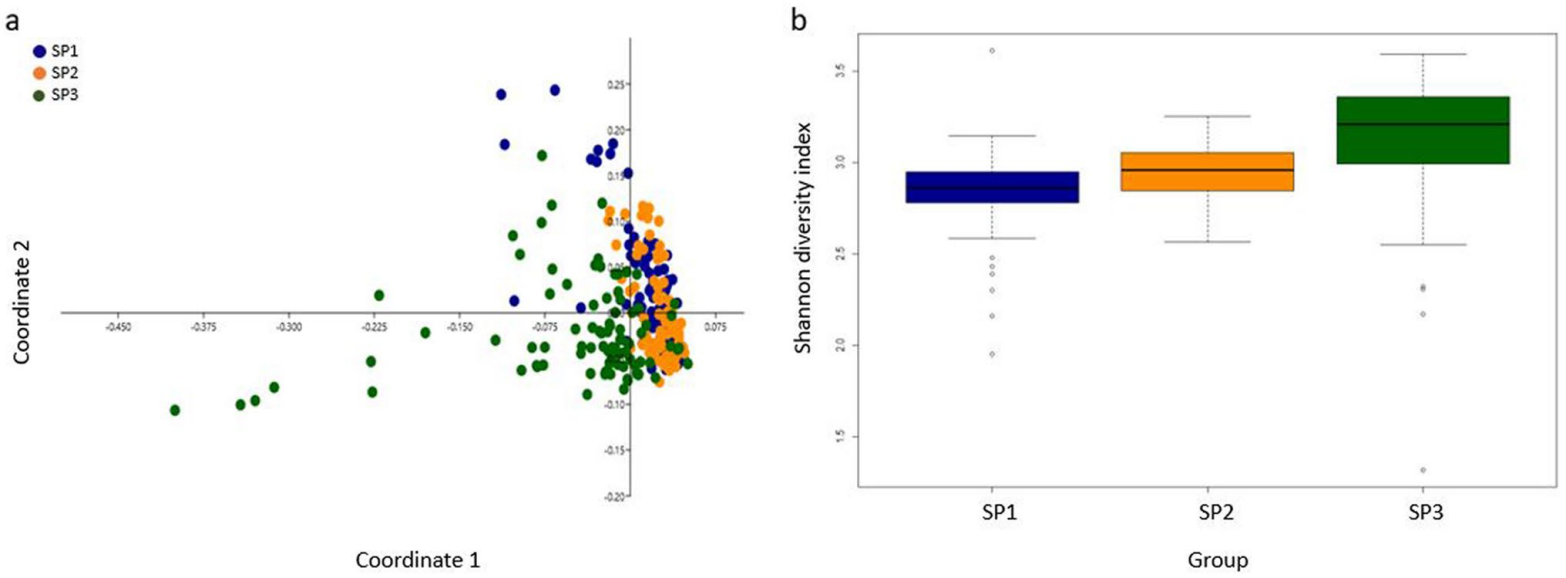
Principal coordinate analysis (PCoA) with Bray–Curtis similarity (**a**) and boxplot of Shannon diversity (**b**) for sampling periods (SP) 1–3.

Faecal bacterial composition in different sampling periods. A PCoA indicated a separation between SPs which was confirmed by ANOSIM analysis (p = 0.0001; R = 0.10). Differences were shown between all SPs; SP1 and SP2 (p = 0.0006; R = 0.04), SP1 and SP3 (p = 0.0003; R = 0.12) and SP2 and SP3 (p = 0.0003; R = 0.16). Moreover, diversity differed (p = 0.0001; R = 0.06) between SPs (Fig. 3) and was lower in SP1 (p = 0.0015; R = 0.05) and SP2 (p = 0.0003; R = 0.11) compared to SP3.

**Figure 4:**
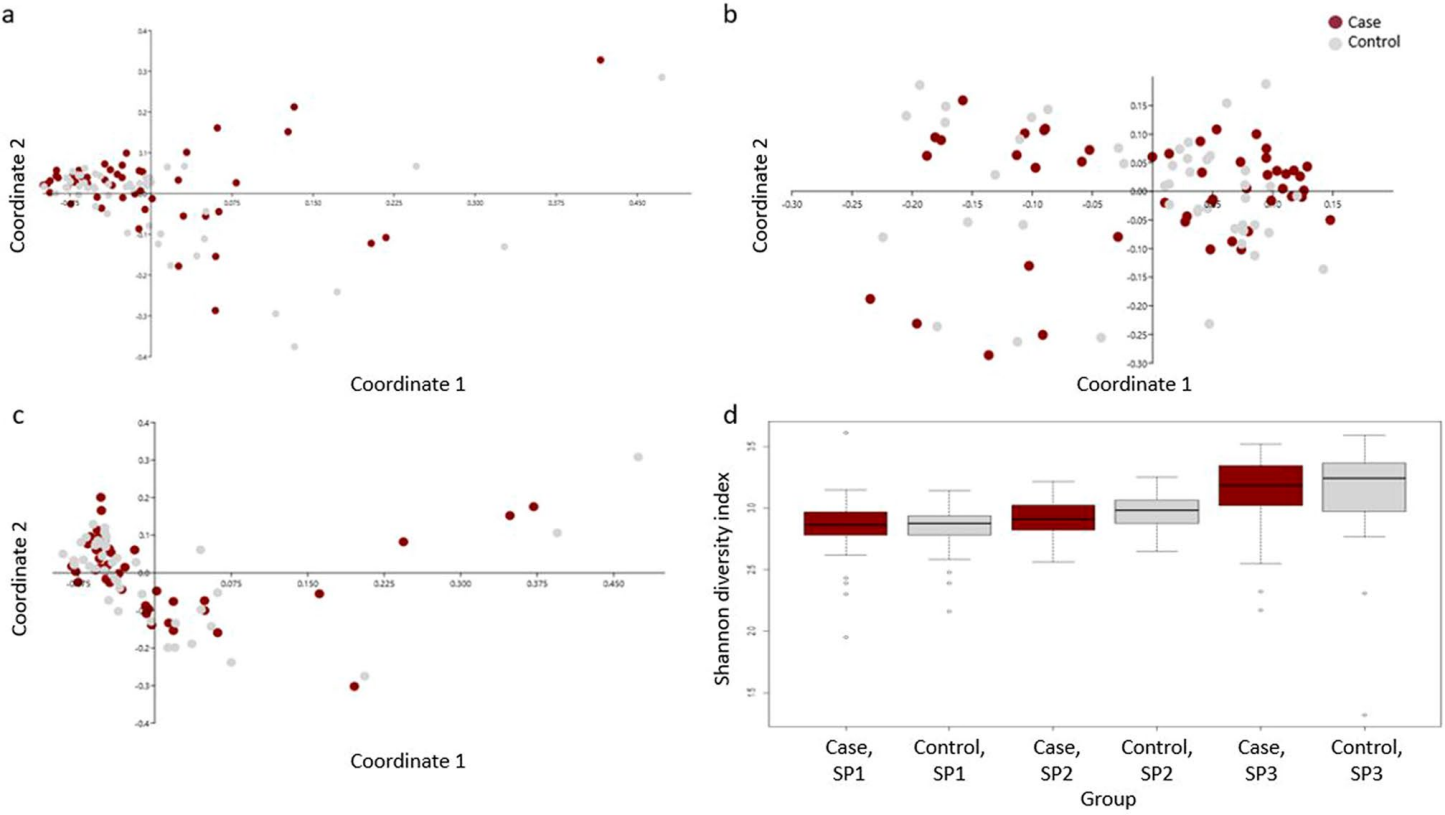
Principal coordinate analysis (PCoA) with Bray–Curtis similarity for horses with free faecal liquid (case) and without free faecal liquid (control) for sampling period (SP) 1–3; SP1 (**a**), SP2 (**b**) and SP3 (**c**), and boxplot of Shannon diversity (**d**) for case and control horses within each SP.

Faecal bacterial composition in case and control horses at each sampling period. Due to the general effect of SP on faecal bacterial composition, comparisons between case and controls were performed within each SP. Visualization using PCoA, did not reveal any separation between case and control horses for SP1 (p = 0.24; R = 0.005) (Fig. 4a), SP2 (p = 0.35; R = 0.002) (Fig. 4b) or SP3 (p = 0.34; R = 0.002) (Fig. 4c). No difference in diversity was shown between case and control horses in any of the SPs (p > 0.05) (Fig. 4d). In parallel, a paired test univariate approach was used to test if specific taxa differed between case and control horses. The univariate analysis identified 14 genera that differed in relative abundance between case and control horses within at least one SP (Table 3), and these genera belonged to the phylum *Bacteroidetes* (n = 2), *Euryarchaeota* (n = 1) and *Firmicutes* (n = 11). On genera level, case horses had a higher abundance of *Alloprevotella* within both SP1 (q = 0.035) and SP2 (q = 0.020), and a tendency for higher abundance in SP3 (q = 0.084) compared to control horses. Case horses displayed a consistent lower abundance of *Bacillus* within SP1 (q = 0.006), SP2 (q = 0.034) and SP3 (q = 0.027), compared to control horses. All taxa that displayed differences in relative abundance of different genera between case and control horses are presented in Table 3.

**Table 3:** Mean relative abundance, % (± SD) of genera in faecal samples of horses with free faecal liquid (case) and horses without free faecal liquid (control) within each sampling period (SP; 1–3).

Taxa level	SP1		q-value	SP2		q-value	SP3		q-value
*(Phylum/Genera)*	Case (± SD)	Control (± SD)		Case (± SD)	Control (± SD)		Case (± SD)	Control (± SD)	
	n = 49	n = 49		n = 48	n = 45		n = 44	n = 42	
*B*^a^*../Alloprevotella*	0.99 ± 0.96	0.62 ± 0.43	0.035	1.20 ± 0.97	0.84 ± 0.42	0.020	0.85 ± 1.05	0.54 ± 0.38	0.084
*B../SP3-e08*	0.89 ± 1.48	0.55 ± 0.60	n.s	0.68 ± 0.66	0.81 ± 1.36	n.s	0.51 ± 0.52	0.36 ± 0.41	0.027
*E*^b^*../Methanobrevibacter*	0.44 ± 0.68	0.77 ± 1.68	n.s	0.76 ± 0.89	0.76 ± 0.16	n.s	0.27 ± 0.62	0.70 ± 2.30	0.017
*F*^c^*../Bacillus*	0.17 ± 0.23	0.23 ± 0.19	0.006	0.19 ± 0.16	0.31 ± 0.37	0.034	0.16 ± 0.19	0.25 ± 0.27	0.027
*F../Blautia*	0.23 ± 0.11	0.20 ± 0.09	n.s	0.21 ± 0.14	0.16 ± 0.10	0.010	0.23 ± 0.11	0.26 ± 0.24	n.s
*F../Clostridium *sensu stricto* 1*	0.19 ± 0.21	0.16 ± 0.12	n.s	0.15 ± 0.11	0.22 ± 0.18	n.s	0.59 ± 0.61	0.35 ± 0.37	0.032
*F../Lachnoclostridium 10*	0.17 ± 0.10	0.24 ± 0.16	0.005	0.30 ± 0.46	0.22 ± 0.13	n.s	0.25 ± 0.13	0.25 ± 0.14	n.s
*F../Lactobacillus*	1.48 ± 7.83	0.35 ± 0.41	n.s	0.27 ± 0.15	0.39 ± 0.41	n.s	0.57 ± 0.67	0.38 ± 0.22	0.033
*F../Marvinbryantia*	0.36 ± 0.16	0.34 ± 0.15	n.s	0.26 ± 0.17	0.26 ± 0.32	n.s	0.28 ± 0.16	0.22 ± 0.17	0.009
*F../Oribacterium*	0.44 ± 0.42	0.43 ± 0.16	0.042	0.38 ± 0.10	0.42 ± 0.14	n.s	0.33 ± 0.13	0.37 ± 0.14	n.s
*F../Roseburia*	0.13 ± 0.08	0.12 ± 0.06	n.s	0.10 ± 0.04	0.11 ± 0.06	n.s	0.12 ± 0.05	0.15 ± 0.26	0.031
*F../Ruminococcaceae UCG-005*	1.81 ± 0.68	1.56 ± 0.50	0.036	1.39 ± 0.32	1.42 ± 1.51	n.s	1.49 ± 0.59	1.28 ± 0.41	n.s
*F../Saccharofermentans*	0.81 ± 0.38	0.78 ± 0.32	n.s	0.75 ± 0.23	0.73 ± 0.19	n.s	0.74 ± 0.23	0.62 ± 0.21	0.017
*F../Solibacillus*	3.63 ± 4.09	5.51 ± 6.40	0.034	3.40 ± 4.05	3.44 ± 3.57	n.s	2.95 ± 3.61	5.19 ± 6.54	n.s

Comparisons based on Wilcoxon matched-pairs signed rank test. False discovery rate (FDR) adjusted p-values are presented as q-values.

^a^*B* = *Bacteroidetes.*

^b^*E* = *Euryarchaeota.*

^c^*F* = *Firmicutes.*

## Discussion

The aim of the present study was to investigate whether there was any difference in faecal microbial composition between horses with and without free faecal liquid (FFL). The results showed a similar overall faecal bacterial composition in case and control horses when visualized in the PCoA and ANOSIM tests did not identify any differences between case and controls. This suggests that there were no general differences in faecal bacterial composition between case and control horses. These results were similar to findings reported by Schoster et al.^9^, who reported only minor differences in faecal microbial composition in horses with and without FFL sampled during spring and autumn. In the present study, case and control pairs were also fed the same forage batches diminishing forage type as a confounding factor. These results indicate that faecal bacterial composition did not differ to any large extent between horses with and without FFL.

Season of year has previously been reported to influence the faecal microbiota composition in horses^11–13^. In the present study, a seasonal effect was observed in the multivariate analysis and hence the data were evaluated separately for the different sampling periods. The three most abundant genera for both case and control horses was *Rikenellaceae RC9 gut group*, followed by *Solibacillus* spp. and *Treponema 2*. *Rikenellaceae RC9 gut group* was reported to be one of the predominant genera in healthy Przewalski horses^14^. This bacterial group has also been found in a higher relative abundance in faeces from non-diarrhoeic compared to diarrhoeic horses^7^. To our knowledge, *Solibacillus* spp. has not previously been reported among the top abundant genera in horse faeces, and information on this bacteria in equine gastrointestinal tract is limited. However, a decreased relative abundance of *Solibacillus* spp. in caecal fluid has been detected in horses with induced laminitis (by infusion of corn starch and oligofructan) compared to control horses (non-infused and non-laminitic)^15^. Relative abundance of *Treponema 2* (and *Treponema* spp.) was lower in horses with compared to without FFL in a previous study^9^, but not in the present where *Treponema* 2 was present in similar relative abundance in case and control horses. *Treponema* spp. has previously been reported as the most abundant genera in Mongolian horses^16^, but also considered among the top 10 most abundant genera in several other horse breeds^17^ and in Przewalski horses^14^. In other populations of healthy horses, *Treponema* spp. was one of the most predominant genera detected, but the abundance of the genera was also shown to be affected by sampling site of faeces as it was higher in the centre of horse faeces compared to on the surface^18^. This may have influenced the results in the present study, even if horse owners were instructed to take the faecal sample from the centre of a fresh pile.

Although faecal microbial composition was in general similar in case and control horses, differences in some low abundant taxa were identified between case and control horses in the paired univariate analysis. This was probably due to the matched pair comparison accounted for in the univariate analysis, but not in the multivariate analysis. The relative abundance for *Alloprevotella* was higher in case compared to control horses in two of three sampling periods. *Alloprevotella* has been detected earlier in the faecal microbiota of sport horses^19^, but was not included in the equine core microbiota (defined as a given genera present in 99.9% of individuals with a relative abundance of at least 0.001%) in a study of endurance horses^19^. Weaning diarrhoea in piglets have been associated with increased proportions of *Alloprevotella*^20,21^, however it is not known if this is a cause or effect of dysbiosis, and pathogenic mechanisms of *Alloprevotella* are not known. In the present study, *Bacillus* spp. was the only genera present in lower relative abundance in case compared to control horses in all SPs. In a previous study investigating the difference in faecal microbial composition between horses with and without colitis, results showed that *Bacillus* spp*.* were one of the predominant genera in the healthy horses but not in horses with colitis^22^. An increase in *Bacillus-Lactobacillus-Streptococcus* (BLS) group were reported in horses adapted to a concentrate diet compared to horses fed a grass-only diet^23^. *Bacillus* spp. are widely distributed in the environment (e.g. in soil and water), but also as part of the normal microbiota in mammals^24,25^. The importance of *Alloprevotella* and *Bacillus* in FFL is not known, but the results of this study indicate that these bacteria could be of interest for further studies on gastrointestinal disturbances (including FFL) in horses. The general faecal bacterial composition and diversity was similar in case and control horses. Previous studies have demonstrated differences in faecal microbiota composition for horses with diarrhoea^7^, colitis^22^ and post-partum colic^6^, compared to healthy controls. In studies of horses with diarrhoea, lower faecal biodiversity, bacterial richness and bacterial evenness was also reported, compared to healthy controls^7,26^. However, for horses with FFL^9^, colitis^22^ or post-partum colic^6^, no difference in faecal microbial diversity was shown compared to their healthy controls. The lack of difference in bacterial diversity in horses with FFL compared to controls in the present study and in a previous study^9^ may indicate that horses with FFL do not have a hindgut bacterial dysbiosis.

Infection with intestinal parasites has been shown to cause loose faecal consistency and diarrhoea in horses^27–30^. Inadequate parasite control is one of the anecdotal suggestions of factors causing FFL in horses. In the current study, the overall endoparasitic burden was low for all horses and there was no difference in parasitic burden between case and control horses. This result is in accordance with previous findings where horses with FFL did not have inadequate parasite control and/or a high parasitic burden^2,3^. Diarrhoea in horses have previously also been reported to be associated with overgrowth of *C. difficile* and *C. perfringens* in the hindgut^31,32^. The results in the present study indicated no association between FFL and growth of *C. difficile* and *C. perfringens* from faecal samples. However, growth of *C. difficile* and *C. perfringens* are known to cause more acute types of diarrhoea and enterocolitis in horses^8,32–36^. None of the horses were reported to have had pyrexia (rectal temperature ≥ 38.2 °C) at the day of sampling or on the two following days. These results indicate that FFL was not associated with ongoing infections, and is in accordance with previous observations of FFL-horses^2,3^.

One reason for not detecting any general differences in faecal bacterial composition between case and control horses could be if there is a difference in the underlying causes of FFL among case horses in the present study. It could be that the microbiota might not be of importance in all cases of FFL. In a previous study^4^, horses showed a large variation in type of clinical symptoms in addition to the two-phased characteristics of faeces during episodes of FFL. Conditions like colic and chronic diarrhea have previously been referred to as single conditions before further investigations showed different aetiologies, leading to different diagnosis and treatments. As FFL is a condition described quite recently, it is possible that several different causes resulting in the same clinical symptom is present. Studies investigating the faecal microbial composition in horses with FFL showing different types of clinical symptoms could add to the knowledge base of FFL and its possible causes.

One limitation of the study was that time from sampling to preparation for analysis may have affected the faecal microbiota composition, either by rapid regrowth of microorganisms and/or microbial contaminants^37^. Due to that participating horses were located all over Sweden and Norway, and sampling periods were the same for all participants, it was not possible to achieve the exact same number of days between sampling and arrival at laboratory. In a study conducted by Beckers et al.^37^, the results showed that equine faecal samples changed in microbial composition rapidly post defecation. Changes included changed diversity and community composition, with increase of several bacterial families including *Bacillaceae*, *Planococcaeae* and *Enterococcaceae*^37^. Although the time from sampling to arrival at the laboratory varied in the current study, samples from each matched pair were taken on the same day and sent in the same postal package resulting in no difference in number of days between sampling and analysis between case and control horse samples.

The results of this study showed no overall differences in faecal bacterial microbiota diversity or composition between stable-matched pairs of horses with and without presence of FFL sampled during three periods. The paired univariate analysis identified differences for *Alloprevotella* and *Bacillus* between case and control horses within all sampling periods. Further studies are required to establish the clinical significance of these low abundant taxa and the overall microbiota in FFL.

## Methods

### Horses

Horses were recruited to the study through an advertisement via web-based channels connected to the Department of Animal Nutrition and Management at the Swedish University of Agricultural Sciences (SLU). The study was designed as a case–control study in 50 private farms with one matched pair of horses at each farm. The farms were located in Norway (n = 20) and Sweden (n = 30). The horses in each pair were kept in the same stable or loose housing system, fed the same batch of forage, and kept in the same or adjacent paddocks. Information on feeding, medical treatment and anthelminthic practices for the last 3 months before the start of the study was collected for all horses. Inclusion criteria for all horses in the study comprised no change in feeds or feeding, no change of stable, being at least 2 years old, no signs of ongoing infection (pyrexia and/or cough) and no ongoing antibiotic treatment during the last 6 months. Additional requirements for control horses included no signs of any gastrointestinal tract conditions during the last 6 months. The definition of a case horse was a horse showing two-phase characteristics of faeces (one solid and one liquid phase), and the definition of a control horse was a horse showing only a solid phase and no separate liquid phase of their faeces. A signed written informed consent was obtained from all horse owners before they entered the study.

### Collection of faecal samples

Non-invasive methods were used for collection of faecal samples. As time of year has been reported to influence faecal microbiota composition in horses^11,12,22^, horse owners were asked to provide a faecal sample from their horses during three sampling periods (SP). The first SP was in October/November 2016 (SP1), the second in December 2016/January 2017 (SP2), and the third in February/March 2017 (SP3). The case–control pair on the same farm was sampled on the same day in all SP. A sampling kit with instructions and materials for collection of faecal samples was provided to all participants just prior to each of the three sampling periods. Faecal samples (approximately 250 g) were collected from the ground or stable floor immediately after observed defecation and placed in double plastic bags. The horse owners were instructed to only sample the part of the faeces that had not touched the ground or floor, to use disposable clean gloves provided in the sampling kit, and to carefully close the bags and send them directly by postal service to the Laboratory at the Department of Animal Nutrition and Management, SLU, Uppsala. Samples were discarded if the time from sampling to arrival at the laboratory exceeded four days, and horse owners then had to provide a new sample within the current sampling period.

### Basic demographics and clinical characteristics

Information on basic demographics and clinical characteristics of the horses was collected through a web-based survey created by use of the software Netigate (Netigate, Stockholm, Sweden). The survey was the same as the one used by Lindroth et al*.* (Lindroth et al., 2020), but for this study only information on horse age, breed, gender and body condition score was used. In addition to survey responses, horse owners were asked to assess the faecal appearance score (FAS; grade 1–7) of their horses according to a template (available in Supplementary Fig. S1) at each sampling period. The same template was used when the samples arrived at the laboratory and were assessed once more by an evaluator (KML) who was blinded to sample identity and horse owner FAS. Horse owners were also asked to provide data on rectal temperature (°C) for their horses on the day of sampling and on the two following days and to provide results from parasitological tests (fecal egg counts) performed during the last 6 months.

### Analysis of *Clostridioides difficile* and *Clostridium perfringens*

At sample arrival to the laboratory each sample was immediately prepared by adding 30 ml buffered peptone water to 30 g sample and running the sample 3 × 30 s at normal speed in a laboratory blender (Seward 3500; Seward Ltd, Worthing, UK). The homogenized samples were then sampled using E-Swabs, which were transferred to the National Veterinary Institute (SVA, Uppsala, Sweden). Cultures for *Clostridium perfringens* were made on fastidious anaerobe (FA) agar (LabM, Lancashire, UK) with 5% defibrinated horse blood. In addition, 1–5 g of the sample was inoculated into 10 ml fastidious anaerobe broth (FAB) (LabM, Lancashire, UK) placed in a 65 °C water bath for 30 min to eliminate vegetative cells and keep the spores and then cultured on FA-agar and incubated in anaerobic jars at 37 °C. The plates were read after 24 and 48 h for growth of *C. perfringens.* For detection of *Clostridioides difficile,* faecal material was streaked on a selective agar medium, Taurocholate Cycloserine Cefoxtin Fructose Agar (TCCFA) containing cycloserine, cefoxitin, fructose, egg yolk and sodium taurocholate (to enhance growth of spores), as described in Båverud et al.^38^. The plates were incubated anaerobically in Oxoid Anaerobic jars at 37 °C and read after 48 and 96 h.

### DNA extraction

Samples were frozen directly on arrival at the laboratory where they were stored at − 80 °C until analysis. For DNA isolation, 180–220 mg of faecal sample was placed in a sterile tube with 0.1 mm zirconium/silica beads (Biospec Products, Bartlesville, Oklahoma, USA) followed by an addition of 1 ml ASL buffer (Qiagen, Hilden, Germany). The samples were then vortexed and homogenization and mechanical disruption of the cell walls was performed with a Precellys homogenizer (Bertin instruments, Montigny-le-Bretonneux, France) 2 × 60 s at the speed 8000 rpm. After centrifugation (10,000*g* for 2 min) the supernatant were transferred to a new sterile tube and centrifuged again at 13,000*g* for 1 min to remove remaining particles. An aliquot (200 µL) of the supernatant were added to another tube containing Proteinase K (15 µL). Lysate (200 µL) were then added before extracting procedure. DNA extraction was performed in a BioRobot EZ1 according to the manufacturer's instructions using the EZ1 Tissue Kit (Qiagen, Hilden, Germany). DNA quantification and quality was assessed with Qubit 3.0 Fluorometer (Life Technologies, Carlsbad, USA). Extracted DNA were sent to Novogene (Tianjin, China) for generation of 16S rRNA gene amplicon libraries and sequencing.

### Generation of 16S rRNA gene amplicon libraries

The V3 and V4 regions of 16S rRNA gene were amplified using primers (341F 5′-CCTACGGGAGGCAGCAG-3′ and 806R 5′-GGACTACNNGGGTATCTAAT-3′). PCR products were purified with GeneJET Gel Extraction Kit (Thermo Fisher Scientific, Massachusetts, USA). Sequencing libraries were generated using NEB Next Ultra DNA Library Prep Kit for Illumina (NEB, Ipswich, USA) and library quality was assessed on the Qubit@ 2.0 Flourometer (Thermo Fisher Scientific, Massachusetts, USA) and Agilent Bioanalyzer 2100 system. The amplicon library was sequenced on an Illumina HiSeq 2500 platform, generating 250 bp paired-end reads. Paired-end reads were merged using FLASH (V1.2.7, http://ccb.jhu.edu/software/FLASH/)^39^ and assigned to each sample according to the unique barcodes. The sequence reads were quality filtered using QIIME (V1.7.0)^40^ and compared with the reference database (Gold database, http://drive5.com/uchime/uchime_download.html) using UCHIME algorithm^41^ to detect and remove chimeric sequences^42^. The reads were clustered using Uparse software (Uparse v7.0.1001)^43^ and OTUs (Operational Taxonomic Units) generated based on 97% sequence homology and OTU representative sequences were then classified taxonomically using the QIIME-based wrapper of the Ribosomal Database Project (Version 2.2)^44^.

### Data processing and statistical analysis

Statistical analyses and data processing were performed in R (R Foundation for Statistical Computing, Vienna, Austria) and PAST^45^. All analysis of faecal microbiota were performed on genera level. Case and control groups were compared for basic demographics, clinical characteristics, FAS and faecal egg count using a Chi^2^-test. For rectal temperature and age, case and control horses were compared using a Wilcoxon matched-pairs signed rank test.

#### Alpha diversity indices of faecal bacteria

Alpha diversity were estimated for 16S rRNA data using the Shannon diversity index (H) to characterize species diversity within groups, taking into account both the number and proportional distribution of taxa in a sample^46^. The α-diversity indices were calculated and displayed using PAST, with data from relative abundance tables. Comparisons between case and control horses and categorical metadata (including sampling period (SP) were analysed. The Wilcoxon matched-pairs signed rank test were used for comparisons between case and control horses.

#### Beta diversity of faecal bacteria

Beta diversity was assessed using Principal coordinates analysis (PCoA) based on Bray Curtis metrics in PAST, and the clustering pattern were statically evaluated using one way- ANOSIM analysis based on 999 permutations^47^ (Clarke, 1993). Univariate analysis was used to assess if specific taxa differed in relative abundance between cases and controls. The univariate analyses were performed on a filtered data set using R (version 3.6. 2). The filtration of data excluded genera that were present in < 20% of the samples or having a mean relative abundance < 0.2%. Differences between case and control horses were tested within each SP and were analysed using the Wilcoxon matched-pairs signed rank test. Statistical differences was accepted at *p* < 0.05 and the Benjamini–Hochberg (B–H) procedure^48^ was used to adjust for multiple comparisons with a false-discovery rate (FDR) of 5%. FDR-adjusted p-values were presented as q-values.

### Consent to participate

A signed written informed consent was obtained from all horse owners before they entered the study.

## Supplementary Information

Supplementary Information.

## Data Availability

The authors confirm that all source data will be deposited on an institutional data repository and made available upon request to the corresponding author.

## References

[1] Zehnder, C. Feldstudie zu Risikofaktoren für den Absatz von freiem Kotwasser beim Freizeitpferd (Doctoral dissertation, lmu) (2009).

[2] Valle E, Gandini M, Bergero D (2013). Management of chronic diarrhea in an adult horse. J. Equine Vet. Sci..

[3] Kienzle E, Zehnder C, Pfister K, Gerhards H, Sauter-Louis C, Harris P (2016). Field study on risk factors for free fecal water in pleasure horses. J. Equine Vet. Sci..

[4] Lindroth KM, Johansen A, Båverud V, Dicksved J, Lindberg JE, Müller CE (2020). Differential defecation of solid and liquid phases in horses—A descriptive survey. Animals.

[5] Gerstner K, Liesegang A (2018). Effect of a montmorillonite-bentonite-based product on faecal parameters of horses. J. Anim. Physiol. Anim. Nutr..

[6] Weese JS, Holcombe SJ, Embertson RM, Kurtz KA, Roessner HA, Jalali M, Wismer SE (2015). Changes in the faecal microbiota of mares precede the development of postpartum colic. Equine Vet. J..

[7] Rodriguez C, Taminiau B, Brévers B, Avesani V, Van Broeck J, Leroux A, Daube G (2015). Faecal microbiota characterisation of horses using 16 rdna barcoded pyrosequencing, and carriage rate of clostridium difficile at hospital admission. BMC Microbiol..

[8] Båverud V, Gustafsson A, Franklin A, Aspan A, Gunnarsson A (2003). Clostridium difficile prevalence in horses, in environment and antimicrobial susceptibility. Equine Vet. J..

[9] Schoster A, Weese JS, Gerber V, Nicole Graubner C (2020). Dysbiosis is not present in horses with fecal water syndrome when compared to controls in spring and autumn. J. Vet. Intern. Med..

[10] Carroll CL, Huntington PJ (1988). Body condition scoring and weight estimation of horses. Equine Vet. J..

[11] Salem SE, Maddox TW, Berg A, Antczak P, Ketley JM, Williams NJ, Archer DC (2018). Variation in faecal microbiota in a group of horses managed at pasture over a 12-month period. Sci. Rep..

[12] Steelman SM, Chowdhary BP, Dowd S, Suchodolski J, Janečka JE (2012). Pyrosequencing of 16S rRNA genes in fecal samples reveals high diversity of hindgut microflora in horses and potential links to chronic laminitis. BMC Vet. Res..

[13] Dougal K, Harris PA, Girdwood SE, Creevey CJ, Curtis GC, Barfoot CF, Newbold CJ (2017). Changes in the total fecal bacterial population in individual horses maintained on a restricted diet over 6 weeks. Front. Microbiol..

[14] Li Y, Zhang K, Liu Y, Li K, Hu D, Wronski T (2019). Community composition and diversity of intestinal microbiota in captive and reintroduced Przewalski’s horse (*Equus ferus przewalskii*). Front. Microbiol..

[15] Moreau MM, Eades SC, Reinemeyer CR, Fugaro MN, Onishi JC (2014). Illumina sequencing of the V4 hypervariable region 16S rRNA gene reveals extensive changes in bacterial communities in the cecum following carbohydrate oral infusion and development of early-stage acute laminitis in the horse. Vet. Microbiol..

[16] Mshelia ES, Adamu L, Wakil Y, Turaki UA, Gulani IA, Musa J (2018). The association between gut microbiome, sex, age and body condition scores of horses in Maiduguri and its environs. Microb. Pathog..

[17] Massacci FR, Clark A, Ruet A, Lansade L, Costa M, Mach N (2020). Inter-breed diversity and temporal dynamics of the faecal microbiota in healthy horses. J. Anim. Breed. Genet..

[18] Stewart HL, Pitta D, Indugu N, Vecchiarelli B, Engiles JB, Southwood LL (2018). Characterization of the fecal microbiota of healthy horses. Am. J. Vet. Res..

[19] Plancade S, Clark A, Philippe C, Helbling JC, Moisan MP, Esquerré D, Mach N (2019). Unrevealing the effects of the gut microbiota composition and function on horse endurance physiology. Sci. Rep..

[20] Hu, P., Wang, F. & Xiao, X. S. Characterization of gut microbiota dysbiosis induced by weaning and diarrhea in Tibetan piglets. *Int. J. Appl. Microbiol. Biotechnol. Res*. 10.33500/ijambr.2019.07.012. (2019).

[21] Wang J, Feng W, Zhang S, Chen L, Tang F, Sheng Y, Peng C (2019). Gut microbial modulation in the treatment of chemotherapy-induced diarrhea with Shenzhu Capsule. BMC Complement. Altern. Med..

[22] Costa MC, Arroyo LG, Allen-Vercoe E, Stämpfli HR, Kim PT, Sturgeon A, Weese JS (2012). Comparison of the fecal microbiota of healthy horses and horses with colitis by high throughput sequencing of the V3–V5 region of the 16S rRNA gene. PLoS ONE.

[23] Daly K, Proudman CJ, Duncan SH, Flint HJ, Dyer J, Shirazi-Beechey SP (2012). Alterations in microbiota and fermentation products in equine large intestine in response to dietary variation and intestinal disease. Br. J. Nutr..

[24] Alou MT, Fournier PE, Raoult D (2016). “Bacillus mediterraneensis”, a new bacterial species isolated from human gut microbiota. New Microbes New Infect..

[25] Lopetuso LR, Scaldaferri F, Franceschi F, Gasbarrini A (2016). Bacillus clausii and gut homeostasis: State of the art and future perspectives. Expert Rev. Gastroenterol. Hepatol..

[26] Elzinga SE, Weese JS, Adams AA (2016). Comparison of the fecal microbiota in horses with equine metabolic syndrome and metabolically normal controls fed a similar all-forage diet. J. Equine Vet. Sci..

[27] Drudge JH (1979). Clinical aspects of *Strongylus vulgaris* infection in the horse. Emphasis on diagnosis, chemotherapy, and prophylaxis. The Veterinary Clinics of North America. Large Anim. Pract..

[28] Owen J, Slocombe D (1985). Pathogenesis of helminths in equines. Vet. Parasitol..

[29] Love S, Murphy D, Mellor D (1999). Pathogenicity of cyathostome infection. Vet. Parasitol..

[30] Gasser RB, Hung GC, Chilton NB, Beveridge I (2004). Advances in developing molecular-diagnostic tools for strongyloid nematodes of equids: Fundamental and applied implications. Mol. Cell. Probes.

[31] Weese JS, Staempfli HR, Prescott JF (2001). A prospective study of the roles of *Clostridium difficile* and enterotoxigenic *Clostridium perfringens* in equine diarrhoea. Equine Vet. J..

[32] Donaldson MT, Palmer JE (1999). Prevalence of *Clostridium perfringens* enterotoxin and *Clostridium difficile* toxin A in feces of horses with diarrhea and colic. J. Am. Vet. Med. Assoc..

[33] Traub-Dargatz JL, Jones RL (1993). Clostridia-associated enterocolitis in adult horses and foals. Vet. Clin. N. Am. Equine Pract..

[34] Madewell BR, Tang YJ, Jang S, Madigan JE, Hirsh DC, Gumerlock PH, Silva J (1995). Apparent outbreaks of *Clostridium difficile*-associated diarrhea in horses in a veterinary medical teaching hospital. J. Vet. Diagn. Investig..

[35] Båverud V (2004). *Clostridium difficile* diarrhea: Infection control in horses. Vet. Clin. Equine Pract..

[36] Diab SS, Songer G, Uzal FA (2013). *Clostridium difficile* infection in horses: A review. Vet. Microbiol..

[37] Beckers KF, Schulz CJ, Childers GW (2017). Rapid regrowth and detection of microbial contaminants in equine fecal microbiome samples. PLoS ONE.

[38] Båverud V, Gustafsson A, Franklin A, Lindholm A, Gunnarsson A (1997). *Clostridium difficile* associated with acute colitis in mature horses treated with antibiotics. Equine Vet. J..

[39] Magoč T, Salzberg SL (2011). FLASH: Fast length adjustment of short reads to improve genome assemblies. Bioinformatics.

[40] Caporaso JG, Kuczynski J, Stombaugh J, Bittinger K, Bushman FD, Costello EK, Huttley GA (2010). QIIME allows analysis of high-throughput community sequencing data. Nat. Methods.

[41] Edgar RC, Haas BJ, Clemente JC, Quince C, Knight R (2011). UCHIME improves sensitivity and speed of chimera detection. Bioinformatics.

[42] Haas BJ, Gevers D, Earl AM, Feldgarden M, Ward DV, Giannoukos G, Methé B (2011). Chimeric 16S rRNA sequence formation and detection in Sanger and 454-pyrosequenced PCR amplicons. Genome Res..

[43] Edgar RC (2013). UPARSE: Highly accurate OTU sequences from microbial amplicon reads. Nat. Methods.

[44] Wang Q, Garrity GM, Tiedje JM, Cole JR (2007). Naive Bayesian classifier for rapid assignment of rRNA sequences into the new bacterial taxonomy. Appl. Environ. Microbiol..

[45] Hammer, Ø., Harper, D. A., & Ryan, P. D. PAST: Paleontological statistics software package for education and data analysis. *Palaeontol. Electron.* **4**(1), 9. https://paleo.carleton.ca/2001_1/past/past.pdf (2001).

[46] Lande, R. Statistics and partitioning of species diversity, and similarity among multiple communities. *Oikos*, 5–13, https://www.jstor.org/stable/3545743 (1996).

[47] Clarke KR (1993). Non-parametric multivariate analysis of changes in community structure. Aust. J. Ecol..

[48] Benjamini, Y. & Hochberg, Y. Controlling the false discovery rate: a practical and powerful approach to multiple testing. *J. R. Stat. Soc. Ser. B (Methodol.)* **57**(1), 289–300, https://www.jstor.org/stable/2346101 (1995).

