# Supplementary material for: Faecal bacterial composition in horses with and without free faecal liquid: a case control study
Source: Sci Rep. 2021 Feb 26;11:4745. doi: 10.1038/s41598-021-83897-4 (PMC7910430; doi:10.1038/s41598-021-83897-4)
Supplement: Supplementary file 1 — Supplementary Information. [file 41598_2021_83897_MOESM1_ESM.pdf]

Faecal bacterial composition in horses with and without free faecal liquid- a case control study

Katrin M. Lindroth[1*], Johan Dicksved[1], Erik Pelve[2], Viveca Båverud[3] & Cecilia E. Müller[1]

[1]Department of Animal Nutrition and Management, Swedish University of Agricultural Sciences, 750 07 Uppsala, Sweden.[2]Department of Anatomy, Physiology and Biochemistry, Swedish University of Agricultural Sciences, 750 07, Uppsala, Sweden.[3]National Veterinary Institute, 751 89 Uppsala, Sweden. *Correspondence to katrin.lindroth@slu.se

Supplement information.

Figure.

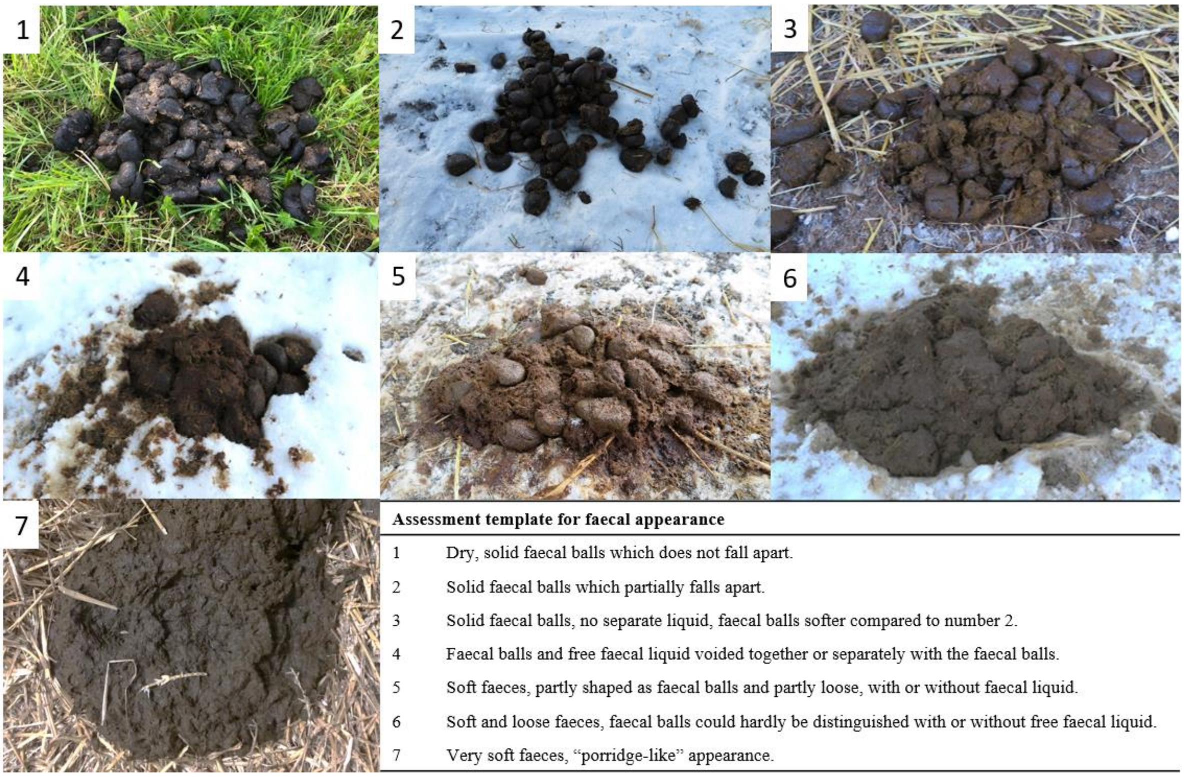

Assessment template for faecal appearance	
1	Dry, solid faecal balls which does not fall apart.
2	Solid faecal balls which partially falls apart.
3	Solid faecal balls, no separate liquid, faecal balls softer compared to number 2.
4	Faecal balls and free faecal liquid voided together or separately with the faecal balls.
5	Soft faeces, partly shaped as faecal balls and partly loose, with or without faecal liquid.
6	Soft and loose faeces, faecal balls could hardly be distinguished with or without free faecal liquid.
7	Very soft faeces, "porridge-like" appearance.

Supplementary Figure S1. Template for assessment of faecal appearance score (FAS). 1 and 7 (photo: K.L. Lindroth), 2-6 (photo: C.E. Müller).

Tables.

Group	Detection level (EPG)	Tapeworm *Anoplocephala perfoliata*	Small strongyles *Cyathostomins*	Large strongyles *Strongylus vulgaris*	Strongyles (not specified) *Cyathostominae/ Strongylus vulgaris*	Roundworm *Parascaris spp.*
Case, n (%)	Not analysed	18 (36)	0 (0)	0 (0)	0 (0)	0 (0)
	Not detected (<50)	32 (64)	44 (88)	50 (100)	44 (88)	50 (100)
	Low (50-200)	0 (0)	4 (8)	0 (0)	3 (6)	0 (0)
	Moderate (201-650)	0 (0)	2 (4)	0 (0)	1 (2)	0 (0)
	High (651-1050)	0 (0)	0 (0)	0 (0)	1 (2)	0 (0)
	Very high (1051-1500)	0 (0)	0 (0)	0 (0)	0 (0)	0 (0)
	Massive (>1500)	0 (0)	0 (0)	0 (0)	1 (2)	0 (0)
Control, n (%)	Not analysed	18 (36)	0 (0)	0 (0)	0 (0)	0 (0)
	Not detected (<50)	32 (64)	44 (88)	50 (100)	40 (80)	50 (100)
	Low (50-200)	0 (0)	3 (6)	0 (0)	3 (6)	0 (0)
	Moderate (201-650)	0 (0)	3 (6)	0 (0)	3 (6)	0 (0)
	High (651-1050)	0 (0)	0 (0)	0 (0)	3 (6)	0 (0)
	Very high (1051-1500)	0 (0)	0 (0)	0 (0)	0 (0)	0 (0)
	Massive (>1500)	0 (0)	0 (0)	0 (0)	1 (2)	0 (0)

Supplementary Table S1. Detection level (EPG) of faecal parasites for case and control horses, as reported by the horse owners. Case and control groups had similar proportions of horses for all variables (P>0.05).

		0 d	1 d	2 d
Case	Min	36.6	36.7	36.6
	Max	38.2	38.2	38.2
	Average ±sd	37.6±0.383	37.6±0.369	37.6±0.325
Control	Min	36.2	36.1	36.1
	Max	38.1	38.2	38.2
	Average ±sd	37.5±0.389	37.6±0.395	37.5±0.345

Supplementary Table S2. Average (±sd) rectal temperature (°C) for case and control horses at the day of sample collection (0 d), and for the one (1 d) and two (2 d) following days after sample collection, as reported by the horse owners. Case and control groups had similar proportions of horses for all variables (P>0.05).
